# Addiction and reward-related genes show altered expression in the postpartum nucleus accumbens

**DOI:** 10.3389/fnbeh.2014.00388

**Published:** 2014-11-05

**Authors:** Changjiu Zhao, Brian Earl Eisinger, Terri M. Driessen, Stephen C. Gammie

**Affiliations:** ^1^Department of Zoology, University of Wisconsin-MadisonMadison, WI, USA; ^2^Neuroscience Training Program, University of Wisconsin-MadisonMadison, WI, USA

**Keywords:** maternal, bonding, reward, addiction, genes, enrichment, nucleus accumbens

## Abstract

Motherhood involves a switch in natural rewards, whereby offspring become highly rewarding. Nucleus accumbens (NAC) is a key CNS region for natural rewards and addictions, but to date no study has evaluated on a large scale the events in NAC that underlie the maternal change in natural rewards. In this study we utilized microarray and bioinformatics approaches to evaluate postpartum NAC gene expression changes in mice. Modular Single-set Enrichment Test (MSET) indicated that postpartum (relative to virgin) NAC gene expression profile was significantly enriched for genes related to addiction and reward in five of five independently curated databases (e.g., Malacards, Phenopedia). Over 100 addiction/reward related genes were identified and these included: *Per1*, *Per2*, *Arc*, *Homer2*, *Creb1*, *Grm3*, *Fosb*, *Gabrb3*, *Adra2a*, *Ntrk2*, *Cry1*, *Penk*, *Cartpt*, *Adcy1*, *Npy1r*, *Htr1a*, *Drd1a*, *Gria1*, and *Pdyn*. ToppCluster analysis found maternal NAC expression profile to be significantly enriched for genes related to the drug action of nicotine, ketamine, and dronabinol. Pathway analysis indicated postpartum NAC as enriched for RNA processing, CNS development/differentiation, and transcriptional regulation. Weighted Gene Coexpression Network Analysis (WGCNA) identified possible networks for transcription factors, including *Nr1d1*, *Per2*, *Fosb*, *Egr1*, and *Nr4a1*. The postpartum state involves increased risk for mental health disorders and MSET analysis indicated postpartum NAC to be enriched for genes related to depression, bipolar disorder (BPD), and schizophrenia. Mental health related genes included: *Fabp7*, *Grm3*, *Penk*, and *Nr1d1*. We confirmed via quantitative PCR *Nr1d1*, *Per2*, *Grm3*, *Penk*, *Drd1a*, and *Pdyn*. This study indicates for the first time that postpartum NAC involves large scale gene expression alterations linked to addiction and reward. Because the postpartum state also involves decreased response to drugs, the findings could provide insights into how to mitigate addictions.

## Introduction

The bond of mother to offspring has been suggested to be the primary social bond in most mammals (Broad et al., 2006) and is a core component of the maternal phenotype. Conserved reward CNS circuitries underlie key life history traits, including sex and eating, and these CNS regions include: medial prefrontal cortex (mPFC), ventral tegmental area (VTA), medial preoptic area (MPOA), and nucleus accumbens (NAC; Kelley and Berridge, 2002). Multiple studies indicate that maternal and social bonding are also regulated by these circuits (Insel, 2003; Numan and Insel, 2003; Burkett and Young, 2012; Olazábal et al., 2013). Among the evidence that mothering involves reward and incentive pathways is that these CNS regions show brain activation in response to offspring cues in humans (Lorberbaum et al., 2002; Bartels and Zeki, 2004; Noriuchi et al., 2008; Strathearn et al., 2008) and rodents (Febo et al., 2005; Ferris et al., 2005; Febo, 2011). Also, rodent mothers will bar press (Wilsoncroft, 1968; Hauser and Gandelman, 1985; Lee et al., 1999) and show a place preference for pups (Mattson et al., 2001, 2003) as they would for other rewarding stimuli. Additionally, drugs of abuse act by coopting natural reward circuitries (Kelley and Berridge, 2002) and mothering mitigates the rewarding properties of addictive drugs, such as cocaine (Mattson et al., 2001, 2003; Mattson and Morrell, 2005; Seip and Morrell, 2007).

Nucleus accumbens’ role in maternal incentive/reward processes has been evaluated from multiple approaches. For example, NAC shows elevated fMRI activation when either human mothers receive cues from children (Lorberbaum et al., 2002; Atzil et al., 2011) or postpartum rats are nursed (Febo, 2011). Dopamine release increases in NAC in rat mothers when they interact with pups [22]. Further, cocaine-induced NAC activation is significantly reduced in rat mothers (Ferris et al., 2005), suggesting reward salience has been shifted to pups whereby pups have become a natural form of addiction. Additionally, pharmacological manipulations and lesions of NAC modulate maternal care (Hansen et al., 1991; Stolzenberg et al., 2007). Pair bonding in prairie voles has many similarities to maternal bonding and pair bonding is linked to NAC function (Young et al., 2011).

Many studies of NAC in natural and drug reward have focused on dopamine and opioid signaling (Pettit et al., 1984; Spanagel et al., 1990; Leone et al., 1991; Peciña and Berridge, 2013), but the human and mouse genome both have more than 20,000 genes and it is becoming increasingly clear that hundreds of genes are linked to drug addiction and reward behaviors (Li et al., 2011; Spanagel, 2013). Further, despite NAC’s links to maternal attachment and addictive processes, no study to date has examined large scale gene expression changes that occur in NAC during this switch in natural reward. In this study, we use microarrays to evaluate large scale gene expression changes that occur in NAC in postpartum females (compared to non-maternal females) using a mouse model. In order to identify genes with connections to reward and addiction, we used our recently developed software tool, Modular Single-set Enrichment Test (MSET), that allows one to evaluate any large scale gene expression dataset against any disease, disorder, pathway, or hand curated database (Eisinger et al., 2013a, 2014; Driessen et al., 2014a). We identified five independently curated databases that maintain lists of genes related to addiction or reward and then tested our significant array results for enrichment against each database. ToppCluster was used to determine whether array results were similar to genes associated with the action of specific drugs or chemicals. Because the maternal state involves an increased risk for mental health disorders (Brockington, 2004), we also used MSET to evaluate whether array results had similarities to mental health disorders using a range of independently curated databases as in recent studies (Eisinger et al., 2013a, 2014; Driessen et al., 2014a). NIH DAVID and ToppCluster were both employed to conduct additional pathway analysis. Weighted Gene Coexpression Network Analysis (WGCNA) identified clusters of genes whose expression may be linked transcriptionally.

## Methods

### Animals

Outbred hsd:ICR female mice (Harlan, Indianapolis, IN, USA) (~70 days of age) were used and procedures were almost identical to those described in detail in our recent microarray study on mPFC (Eisinger et al., 2014). In brief, mice were nulliparous when obtained and half were housed with breeder males for a 2 week mating period, while the other half were housed with age-matched female littermates to minimize isolation-induced stress. After mating, all females were housed individually until parturition (postpartum day 0) so that all subjects had a similar social environment. We have previously used these groups to identify gene expression changes that correspond to collective experiences (pregnancy, parturition, and postpartum) that generate the maternal phenotype (Zhao et al., 2012b; Eisinger et al., 2013b; Driessen et al., 2014a,b). *Ad libitum* breeder chow (Harlan) and water were provided along with precut nesting material. Polypropylene cages were changed weekly prior to parturition, after which cages were not changed again until dissection. On day 0, litters were culled, if necessary, to standardize litter size to 11. All subjects were kept on a 12:12 light:dark cycle with lights on at 6:00 CST. All procedures followed guidelines set by the National Institutes of Health Guide for the Care and use of Laboratory Animals, and were approved by the University of Wisconsin Animal Care and Use Committee.

### Tissue collection and RNA extraction

**Figure 1:**
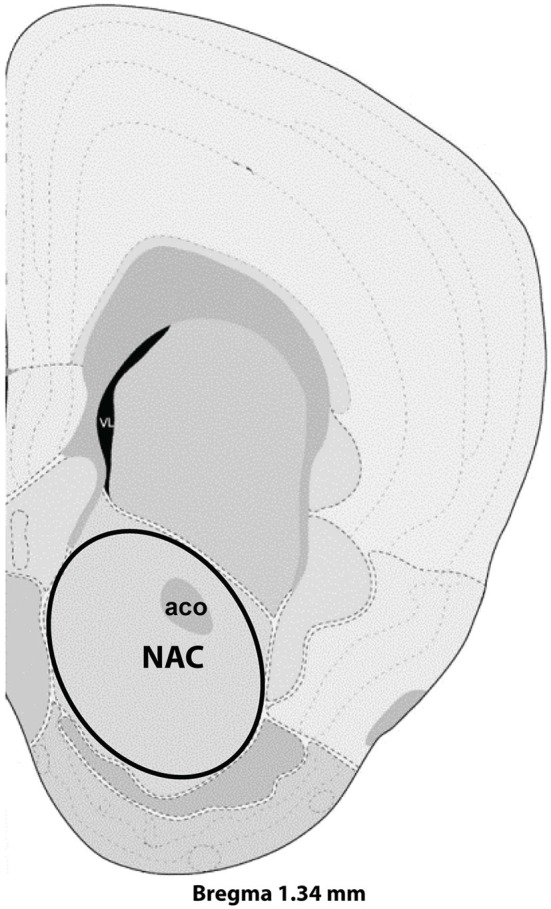
**Representative section with NAC dissection for microarray analysis**. Distance from Bregma in the rostrocaudal plane is indicated. Modified from the Allen Mouse Brain Atlas (reference atlas version 1, 2008). Abbreviations: aco, anterior commissure; NAC, nucleus accumbens.

Virgin and postpartum females were lightly anesthetized with isuflurane and decapitated between 9:00 and 12:00 CST on postpartum Day 7. Brains from age-matched virgin and postpartum females were collected on the same day and dissections were alternated between groups. After decapitation, vaginal lavage allowed for determination of estrous state. To minimize effects of estrous cycling on gene expression (Romano et al., 1988; Arosh et al., 2002), only diestrous virgins were used in the microarray experiment. Brains were frozen in isopentane, stored at −80°C, sectioned via cryostat (Leica CM1850, Bannockburn, IL, USA) at 200 micrometer thickness, and NAC collected via a micropunch technique (Makino et al., 1994) using a Brain Punch Set (Stoelting, Wood Dale, IL, USA) under a dissecting microscope. Nucleus accumbens tissue was collected from Bregma 1.54 mm to Bregma 1.045 mm as shown in Figure 1 and included both core and shell regions of NAC. Samples were collected from 10 postpartum females and 10 virgin females, and were subsequently stored at −80°C until RNA extraction. RNA extraction and purification was exactly as recently described (Eisinger et al., 2014) and involved the Aurum Total RNA Fatty and Fibrous Tissue Kit (Bio-Rad, Hercules, CA, USA) and the NanoDrop 2000 spectrophotometer (Thermo Scientific, Wilmington, DE, USA) and RNA was stored at −80°C until further processing. For the microarray studies, six mice from each group were randomly selected for analysis as previous microarray studies indicate six per group is sufficient to detect differences in treatment. An N of 10 per group was used for follow up quantitative PCR (qPCR) analysis.

### High-density oligonucleotide array hybridization

Microarray analysis was performed with the GeneChip Mouse Gene 2.0 ST Array (Affymetrix, Santa Clara, CA, USA) with targets derived from total RNA from NAC. Approaches were identical to those recently described (Eisinger et al., 2014) and included the Ambion GeneChip WT Expression Kit (Ambion, Austin, TX, USA), the Affymetrix WT Terminal Labeling Kit (Affymetrix), and an Affymetrix GC3000 G7 Scanner. Data were extracted and processed in the Affymetrix Command Console v. 3.1.1.1.229 and cDNA synthesis, fragmentation, labeling, array hybridization, staining, and scanning were performed by the Gene Expression Center at the University of Wisconsin-Madison as in previous studies (Eisinger et al., 2013b, 2014; Driessen et al., 2014a).

### Probeset level summarization and microarray statistical analysis

Probe logarithmic intensity error (PLIER) algorithm in Affymetrix Expression Console, build 1.2.1.20 was used for probeset level summarization and normalization. The BioConductor package limma v3.14.4 was used to perform statistical analysis. The nominal PLIER *p*-value of 0.01 was used for analysis as in previous studies (Saul et al., 2012; Eisinger et al., 2013b, 2014; Driessen et al., 2014a) and a *p*-value of 0.03 was also used for additional data mining. Fold change was calculated for each gene as the ratio of the limma-calculated average maternal expression coefficient divided by average virgin expression coefficient.

### Modular Single-set Enrichment Test (MSET)

**Figure 2:**
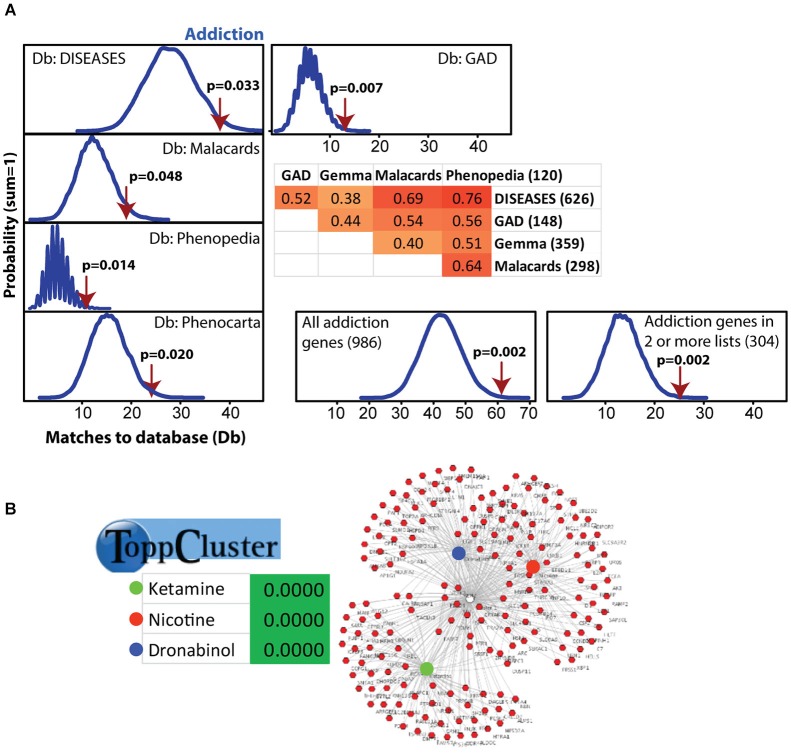
**Modular Single-set Enrichment Test evaluation of enrichment for addiction related gene sets within significantly altered genes in maternal NAC. (A)** Y-axis represents the probability of X matches to database appearing in a randomly generated set of simulated results from the microarray background. The red arrow shows how many matches were found in the actual significant postpartum NAC expression changes and where that number falls on the probability density distribution. The enrichment *p*-value is derived from the number of simulated results that contained at least as many matches to database as the actual results. The number of genes within each database and the overlap (smaller sized list divided by larger list) is provided. **(B)** ToppCluster results showing enrichment of significant postpartum NAC genes with genes associated with three drugs, ketamine, nicotine, and dronabinol. Graph on right shows the general pattern whereby some genes are only linked to one drug, but others are linked to two or three.

MSET was used to test significant microarray results for enrichment of gene lists with multiple databases. To evaluate links to addiction, we identified five independent online gene-disease association databases, including the HuGE Navigator’s Phenopedia (Yu et al., 2010), the DISEASES database by the Novo Nordisk Foundation Center for Protein Research at the University of Copenhagen (Pletscher-Frankild et al., 2014), the NIH’s Genetic Association Database (GAD; Becker et al., 2004), the Weizmann Institute of Science’s MalaCards compendium (Rappaport et al., 2013), and Gemma’s Phenocarta (Zoubarev et al., 2012). Addiction research often focuses on drugs of abuse and within each database multiple categories are provided, including general addiction or chemical dependency or genes known to be linked to different types of addiction, including cocaine, methamphetamine, heroin, nicotine, and alcohol. Within each database there are some overlaps of the different subsets. For Phenocarta, we kept together genes linked to all types of addiction (total gene number is 359), but for the other four we separately analyzed alcohol because these lists were often larger than all other lists combined (range from 337 to 675). Further, for GAD, the nicotine list alone was 2945 genes, so this was also analyzed separately. The level of overlap between the different addiction databases is shown in Figure 2. As an additional analysis, we created a database that included all unique genes from each of the five addiction databases (*n* = 986) and created an additional database for genes that showed up in two or more lists (*n* = 304). We also used MSET to test for enrichment against various mental health disorders and diseases as we recently performed using gene expression results from other maternal brain regions (Eisinger et al., 2013a, 2014; Driessen et al., 2014a).

Modular Single-set Enrichment Test uses a randomization testing algorithm to assess overrepresentation of any database (e.g., any mental health disorder, any disease) within significant microarray results. It generates a null distribution of expected number of matches to a given database of interest in randomly generated results built by sampling without replacement from the microarray background and this has been described in detail (Eisinger et al., 2013a). In this study, we tested enrichment within microarray results with *p*-values less than 0.01 using 10,000 randomized sets of results. Current and previous evaluations indicate that almost all of the genes in this range can be confirmed via qPCR and are biologically relevant (Saul et al., 2012; Zhao et al., 2012b; Eisinger et al., 2013b, 2014). In this study we also confirmed a number of genes with *p*-values less than 0.03, but greater than the *p* < 0.01 cutoff and for additional data mining we ran MSET using a *p-value* cutoff of 0.03. As with any array, there can be a small number of false positive and in the present study, for example, we could not confirm *Grm7* changes. MSET works with larger datasets and in this study the removal of one or two genes (from 1052 annotated genes in the microarray) when compared with databases with hundreds of genes had little contribution to the overall *p*-value. Therefore, we report here the *p*-value using all 1052 genes, but remove any unconfirmed gene from further analysis.

### Analysis with ToppCluster and NIH DAVID

Microarray targets with *p*-values less than 0.01 were used for both ToppCluster (Kaimal et al., 2010) and NIH DAVID analysis (Huang da et al., 2009). The default statistical *p*-value analysis was used for both. While some databases are shared between the two, each also provides unique analysis. For example, ToppCluster allows for analysis of enrichment against genes associated with various drug actions, and this analysis was used in this study.

### WGCNA and transcription factor analysis

Weighted Gene Coexpression Network Analysis was used to identify modules of genes whose expression changes are highly correlated to one another. R software was used for all WGCNA analysis (Zhang and Horvath, 2005; Langfelder and Horvath, 2008) and the array *p*-value cutoff was <0.01. To generate a weighted network of genes (nodes) and their expression correlations (edges), correlations were raised to a soft thresholding power β, chosen such that the network approximates a model of scale-free topology (*R*^2^ > 0.8), which is a necessary assumption for WGCNA accuracy. Using unsupervised hierarchical clustering, a minimum module size of 30 genes, and a threshold setting for merging modules of 0.25, WGCNA identified two modules. The modules were exported as a Cytoscape network file, which was manually trimmed to consist only of transcription factor nodes and their gene-to-gene correlations. The finalized transcription factor module was visualized with Cytoscape v3.0.1.

### Quantitative real-time PCR

Polymerase chain reaction was performed on genes of interest (*n* = 10 per group) in order to confirm expression changes detected by microarray analysis. Two stable reference genes were used to normalize relative expression results of genes of interest; Tyrosine 3-monooxygenase/tryptophan 5-monooxygenase activation protein, zeta polypeptide (*Ywhaz*), and peptidylprolyl isomerase A (*Ppia*) (also known as CycA). Control genes were chosen because they have been shown by others to act as reliable control genes for qPCR in the rodent brain (Bonefeld et al., 2008; Nelissen et al., 2010) and have been found by us to be reliable markers for the postpartum brain (Zhao et al., 2012a,b; Eisinger et al., 2013b, 2014; Driessen et al., 2014b).

A SuperScript III First-Strand Synthesis System for RT-PCR (Invitrogen, Carlsbad, CA, USA) was used to reverse transcribe 100 ng of RNA to cDNA in an Eppendorf MasterCycler Personal PCR Machine (Eppendorf, Hamburg, Germany) with poly-T 20mer primers. The thermal profile used is as follows: an initial melting step of 95°C for 30 s, followed by 40 cycles of a 5-s 95°C melt, a 20-s 58°C annealing step, and a 20-s 72°C elongation step. A melt curve was performed from 60–95°C at 5-s 0.5°C increments to confirm specificity of primer binding, and relative expression values were calculated with REST 2009 (Pfaffl et al., 2002).

## Results

### Genes with altered expression in maternal NAC

High density oligonucleotide microarray (41,346 probes) was performed on NAC and results were analyzed with the PLIER algorithm. Altered gene expression was found in postpartum NAC for 1260 probes (1052 unique, annotated genes) using a *p* < 0.01 cutoff and for 2740 probes (2164 unique, annotated genes) using a *p* < 0.03 cutoff. The full set of results is available in Supplementary Table 1. All microarray results, including CEL files, have been uploaded to NCBI’s Gene Expression Omnibus (Accession number: GSE62258).

### MSET and ToppCluster analysis for enrichment of gene sets associated with addiction

**Figure 3:**
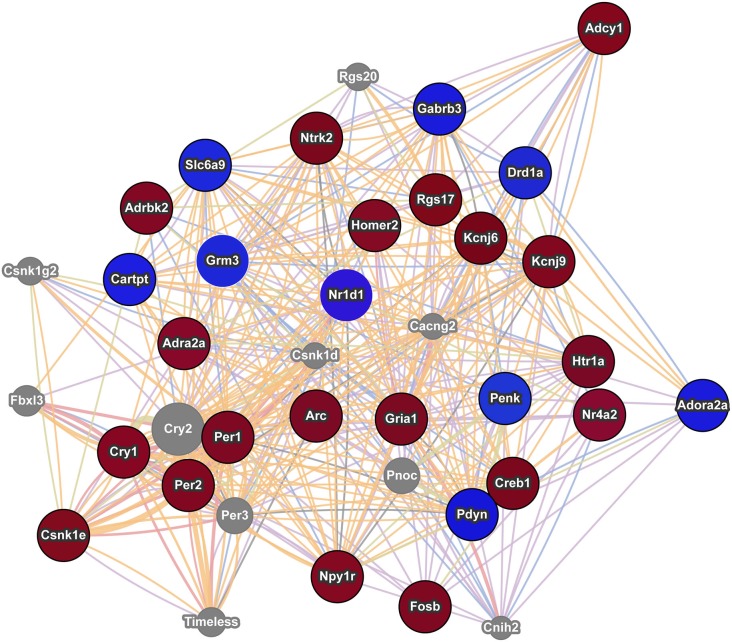
**Interaction network of addiction related genes with altered expression in postpartum NAC**. The red circles indicate upregulation and blue circles indicate downregulation of genes within significant maternal NAC expression results. Results were visualized as an interaction network with genes in gray circles added by GeneMania due to interactions with specified genes. *Nr1d1* was added to network due to recent studies linking it to addiction pathways and because of its multiple interactions with known addiction related genes. The nature of the interaction data linking any two genes is encoded by color (blue lines = colocalization, purple lines = coexpression, red lines = physical interactions, light green = shared protein domains, and orange lines = predicted interaction). Distance between genes is proportional to the strength of evidence for their interaction.

Modular Single-set Enrichment Test was used to assess enrichment for genes associated with addiction within genes displaying altered expression (*p* < 0.01) in maternal NAC. Details on the databases are provided in the Methods section. As shown in Figure 2A, the postpartum NAC showed enrichment for addiction and reward related genes in five of five independent datasets (*p* < 0.05). Information on the number of genes in each list and the extent of overlap between lists is provided in Figure 2A. Furthermore, enrichment was found when either all addiction related genes were pooled to create a novel database (*N* = 986; *p* < 0.01) or when only genes in two or more lists were pooled to create a novel database (*N* = 304; *p* < 0.01). Notable genes found in more than one database were: *Per1*, *Per2*, *Penk*, *Homer2*, *Creb1*, *Ntrk2*, *Nr4a2*, and *Fosb*. Further, we ran MSET using an array *p* < 0.03 cutoff (see rationale above) and also found significant enrichment for all addiction/reward datasets (see Supplementary Table 2). Among the interesting genes with *p*-values between 0.01 and 0.03 were: *Cartpt*, *Adcy1*, *Npy1r*, *Htr1a*, *Drd1a*, *Gria1*, and *Pdyn*. Supplementary Table 2 provides a list of all genes (*N* = 113) with *p*-values less than 0.03 that were associated with one or more addiction database. When analyzed with ToppCluster, significant postpartum NAC genes showed a significant overlap with genes involved in the action of drugs of abuse, including nicotine, ketamine, or dronabinol (a synthetic cannabinoid analog; *p* < 0.001 each; Figure 2B). A GeneMania diagram of interactions of a subset of addiction/reward related genes in postpartum NAC is shown in Figure 3. Although *Nr1d1* was not in any of the five addiction lists, it was included here because it has recently been implicated in addiction processes (see Section Discussion below), its expression is significantly downregulated in NAC (see below), and it is centrally connected to addiction/reward related genes.

Because alcohol addiction related genes had large datasets, these were analyzed independently, but were commonly just above significance (*p* = 0.06, 0.09, 0.06, and 0.34 for the four available databases; see Supplementary Table 3). Also, the nicotine related database from GAD was large (*N* = 2945), so this was analyzed independently and was significantly linked to maternal NAC expression (*p* < 0.001; see Supplementary Table 3).

### MSET analysis for enrichment of gene sets associated with mental health disorders and other diseases

**Figure 4:**
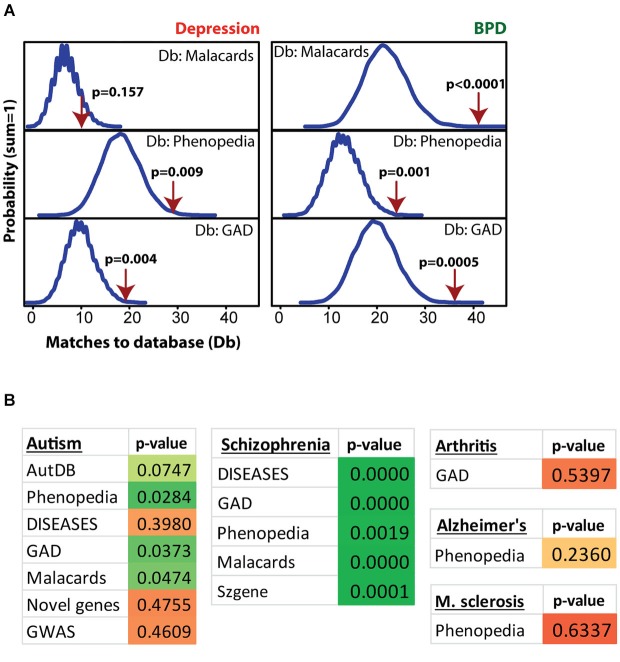
**Modular Single-set Enrichment Test evaluation of enrichment for mental health related gene sets within significantly altered genes in maternal NAC. (A)** Y-axis represents the probability of X matches to depression and BPD databases appearing in a randomly generated set of simulated results from the microarray background. The red arrow shows how many matches were found in the actual significant postpartum NAC expression changes and where that number falls on the probability density distribution. The enrichment *p*-value is derived from the number of simulated results that contained at least as many matches to database as the actual results. **(B)** Modular Single-set Enrichment Test *p*-values for significant postpartum NAC genes against databases for autism, schizophrenia, arthritis, Alzheimer’s disease, and multiple sclerosis.

Figure 4 shows MSET analysis for genes linked to depression, bipolar disorder (BPD), autism, schizophrenia, arthritis, Alzheimer’s and multiple sclerosis. Details on the databases are provided in the Methods section. Postpartum NAC array genes (*p* < 0.01) showed consistent significant enrichment for depression (two of three databases), BPD (three of three databases), and schizophrenia (five of five databases). For autism, only three of seven databases showed significant enrichment. A list of postpartum NAC genes with links to mental health disorders is provided in Supplementary Table 4. For arthritis, Alzheimer’s disease, and multiple sclerosis, no enrichment was found (Figure 4).

### Pathway analysis with NIH DAVID, ToppCluster, and MSET

NIH DAVID and ToppCluster identified enrichment for RNA binding and NIH DAVID further found enrichment for genes involved with CNS development/differentiation, transcriptional regulation, and circadian rhythms. A list of 41 postpartum NAC genes with connections to various aspects of CNS development, such as axon growth, axonogenesis, neurogenesis, and neuron differentiation is provided in Supplementary Table 5. Using MSET and a database of known transcriptional regulators in mice, the Animal Transcription Factor Database, we found significant enrichment of postpartum NAC for transcriptional regulators (*p* < 0.00001). A total of 128 genes that contribute to transcriptional regulation were found within significant postpartum array results (~12% of all genes). A list of these genes along with additional transcriptional regulation genes from NIH DAVID is provided in Supplementary Table 5. NIH DAVID analysis also revealed enrichment for acetylation (172 genes), histone deacetylation complex (genes included: *Hdac4*, *Hdac5*, *Nrip1*, *Morf4l1*, *Sap30*, and *Suds3*) and histone acetyltransferase complex (genes included: *Ep300*, *Taf12*, *Yeats4*, *Eny2*, and *Morf4l1*) (see Supplementary Table 5). KEGG pathway analysis via NIH DAVID identified enrichment for circadian rhythm and the genes identified were: *Csnk1e*, *Cry1*, *Npas2*, *Nr1d1*, *Per1*, and *Per2*.

### qPCR analysis of a subset of genes

**Figure 5:**
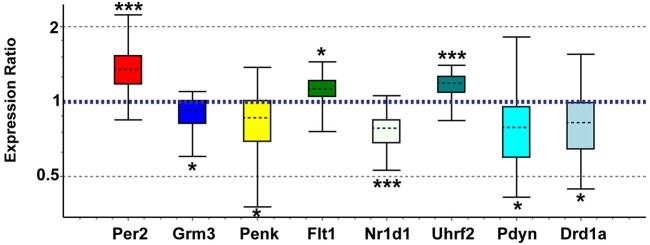
**Quantitative real-time PCR confirmation of expression changes for genes of interest in maternal NAC compared to virgin**. Relative expression distribution (Y-axis) represented as a ratio of postpartum vs. virgin (*n* = 10 per group) normalized against two references genes, *Ppia* and *Ywhaz*, and shown by box-and-whisker plots as medians (black dashed lines), interquartile ranges (boxes), and ranges (whiskers). Ratios over 1 indicate genes that are more highly expressed in postpartum NAC than in virgin, while ratios less than 1 indicate genes with lower expression in postpartum females. *p < 0.05; ***p < 0.001.

Genes of interest for real-time qPCR confirmation were selected based on biological importance and concordance with recent studies in other brain regions. We confirmed array expression changes (*p* < 0.01) via qPCR (all *p* < 0.05) for *Grm3*, *Flt1*, *Penk*, *Nr1d1*, *Uhrf2*, and *Per2* and PCR direction was always the same as array direction (Figure 5). The array decreases in *Fabp7* in NAC were confirmed in a separate study (unpublished observations). We did not confirm *Grm7*, *Oprk1*, or *Dscam* (*p* > 0.05). We also confirmed via qPCR (*p* < 0.05) expression changes for genes with array *p*-values between 0.01 and 0.03 that have strong connections to addiction and reward, including *Pdyn* (array *p* = 0.026) and *Drd1a* (array *p* = 0.016; Figure 5). Further, elevated *Oxtr* (array *p* = 0.07) and decreased *Pde4b* (array *p* = 0.043) were confirmed as significant in postpartum NAC increases using qPCR (*p* < 0.05) (not shown). Primer information on tested genes can be viewed in Supplementary Table 6.

### WGCNA analysis

**Figure 6:**
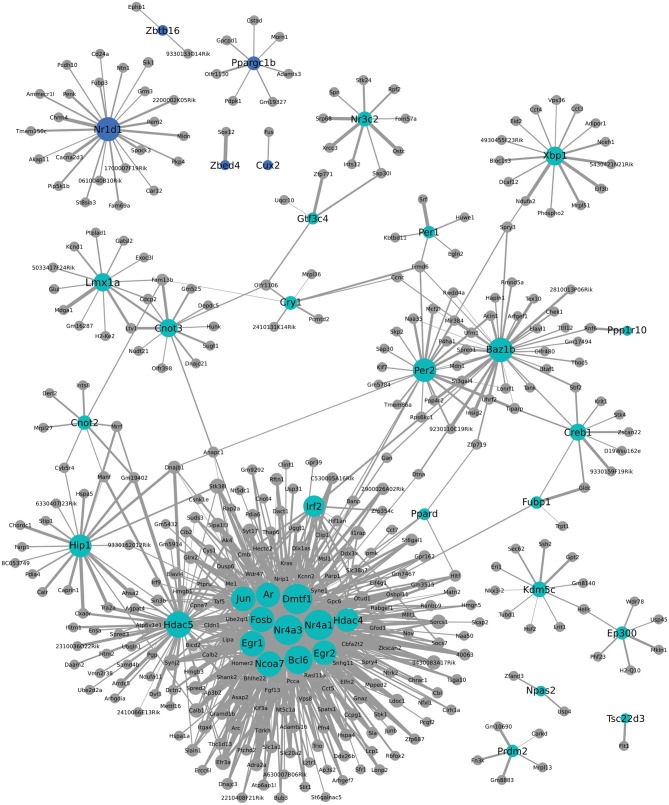
**Weighted Gene Coexpression Network Analysis network of transcription factors and genes with highly correlated expression changes in postpartum NAC**. Within the gene module identified by WGCNA as having highly correlated expression, transcription factors (light blue or blue circles with black labels) are visualized in relation to other module genes. The significance of the correlation is indicated by the width of the line, with thicker lines reflecting more significant correlation.

We additionally used WGCNA to identify modules of genes that cluster together based on co-expression. This approach can provide indirect insight into transcriptional regulatory networks. To investigate a possible role for transcription factors in the coordinated expression of this gene network, expression correlations for transcription factors found within two modules were visualized in Cytoscape. As seen in Figure 6, *Nr1d1* (in blue module) may have an important role in transcriptional regulation of some postpartum genes, including *Penk* and *Grm3*. Other circadian transcriptional regulators, including *Cry1*, *Per1*, and *Per2* (turquoise module), and genes they may regulate in the postpartum state are also shown in Figure 6. A subset of transcriptional regulators, including *Fosb*, *Jun*, *Egr1*, *Ar*, *Dmtf1*, *Nr4a3*, *Nr4a1*, *Ncoa7*, *Egr2*, *Hdac5*, and *Irf2* are themselves clustered together and are indicated to act as a network to regulate ~100 postpartum genes (Figure 6).

## Discussion

In this study we find evidence that the large-scale gene expression changes in NAC in postpartum females include numerous genes linked to addiction and reward. The finding is consistent with NAC’s role in addiction or reward/incentive related behaviors and that offspring are highly rewarding to mothers (see Section Introduction). The novelty of this study is the identification of over a hundred addiction/reward-related genes that are recruited and likely promote the emergence of a new natural reward, the offspring. The finding of multiple postpartum NAC genes that are involved in CNS plasticity and development is consistent with findings in other maternal brain regions and suggests the maternal brain may be a developmental endpoint. That a number of genes with altered expression are involved in transcription regulation provides a means for understanding how the maternal NAC is produced as does the WGCNA analysis that locates coexpression modules. The links of maternal NAC expression to some mental health disorders is similar to recent findings in other regions and has multiple implications as discussed below.

### Genes with altered expression in postpartum NAC linked to addiction and reward

One of the most striking findings of the present study was the high enrichment of significant postpartum NAC genes with multiple databases with genes linked to addiction, dependency, and reward. Further when all genes from all the databases were brought together or genes that appeared in two or more lists were brought together to make novel databases, the significant enrichment was also found using MSET (Figure 2A). Although *p* < 0.01 is a typical array cutoff, we confirmed via qPCR genes with *p*-values less than 0.03, but greater than 0.01, including *Drd1a* and *Pdyn*. We also confirmed decreased *Pde4b* and elevated *Oxtr* via qPCR although the respective array *p*-values were 0.043 and 0.07. *Oxtr* is of interest because of previous work showing a positive association of the receptor in NAC to maternal care (Olazabal and Young, 2006). Together, these findings and those of other arrays (Saul et al., 2012; Zhao et al., 2012b) suggested that a number of genes with *p*-values above 0.01 have biologically meaningful expression changes. To provide a broader survey of large scale changes we also tested for enrichment using MSET for genes with array *p* < 0.03. Again, significant enrichment of postpartum NAC genes with databases for addiction was found and a list of those genes is provided in Supplemental Table 2. The addiction/reward databases used in this study are independently curated and it is possible that the evidence for some of the genes is weak and they may prove to have little contribution to reward processes. However, it is also possible that some lesser known genes are included in these lists that do play an important role in addiction/reward processes. If we and others continue to find associations with such genes, it is possible that these previously unnoticed, but important genes, will now receive attention.

The idea of the postpartum NAC being in a new natural reward state was supported by ToppCluster analysis that found significant links of postpartum NAC genes with genes associated with either nicotine, ketamine, or dronabinol (a synthetic cannabinoid analog; Figure 2B). Modular Single-set Enrichment Test analysis of the one nicotine (GAD) database was significant, but none of the four databases for alcohol were significant. Although it is speculative, aspects of nicotine addiction and dependency appear to have greater similarities to the maternal NAC than does alcohol addiction.

Using an array *p*-value cutoff of 0.03, we identified over a hundred genes that are related to reward/incentive/addiction related processes. One approach to understand this multitude of genes is to evaluate each gene independently and to determine how they interact with one another. Some genes have been well studied in terms of addiction and/or maternal function. For example *Cartpt* (also known as Cart) derives its name from its response to cocaine and amphetamine and is implicated in reward processes (Hurd et al., 1999; Rogge et al., 2008). Further, Cart expression in NAC is modulated by pup cues in rat mothers (Mattson and Morrell, 2005). *Creb1* (also known as Creb) and variants of *Fosb*, including delta Fosb, are involved in addictive responses (McClung and Nestler, 2003) and maternal care is disrupted in *Fosb* mutant mice (Brown et al., 1996). *Drd1a* (also known as Drd1) is involved in addictive responses (Comings et al., 1997; Le Foll et al., 2009) and maternal care in rats (Parada et al., 2008) and humans (Mileva-Seitz et al., 2012). Genemania provides gene connections based on numerous datasets, including coexpression and physical interactions. As shown in Figure 3, some interesting links occur between these genes and Genemania identifies additional genes (in gray) that may act as mediators between the focus genes. *Nr1d1* (also known as rev erb alpha) was not in any of the five addiction databases, but it is significantly downregulated in maternal NAC and recent studies are beginning to suggest an important role for this gene in addiction and reward related behaviors (Belluardo et al., 2005; Wang et al., 2008; Piechota et al., 2012; Wongchitrat et al., 2013). *Nr1d1* is a transcriptional factor integral to function of circadian rhythm genes and to mental health disorders (see below). As seen in Figure 3, *Nr1d1* is tightly linked to other addiction related gene and as highlighted by WGCNA analysis, it may be a critical transcriptional regulator in the maternal brain. It is possible that *Nr1d1* may provide a newer avenue for understanding the strong link between mental health disorders and the likelihood of addiction, but this would need to be addressed in subsequent studies.

Nucleus accumbens is a central region in reward related behaviors, but it is part of a network and it will be valuable to understanding of how modifications of NAC are coordinated with other regions. Further, NAC includes both core and shell regions (Kelley and Berridge, 2002) and a detailed understanding of how gene expression changes in NAC are manifested in terms of subregions will be important for future work on natural shifts in reward. Further, how genes are expressed in given cell types is of great importance. For example, while *Fabp7* is found mostly in glial cells along with neural progenitors (Matsumata et al., 2012; Yun et al., 2012),* Penk* is clearly neuron-specific. A detailed understanding of gene expression changes within cell subtype will also be key to moving our understanding forward.

In this study we avoided any maternal testing as tests themselves can alter gene expression. All pups were healthy at the time of tissue collection, indirectly indicating that maternal care, including nursing, was sufficient. However, variance in levels of maternal care has been documented in rats and mice (Champagne et al., 2003, 2007) and even with seemingly similar maternal profiles, the reward responding can differ significantly among mothers at day 10 in rats (Mattson et al., 2003). Reward responding to pups is high on day 8 in rats and shifts away from offspring at day 10 and beyond as they mature (Mattson et al., 2001). In this study we evaluated female mice on postpartum day 7 when it would be expected that reward responding to pups was still high. Because we did not test for reward responding in this study, we cannot draw any direct conclusions, but suggest that the composite brain changes we observed in postpartum NAC correspond to the composite changes that occur in reward responding at this time (even though individual differences may occur). It would be of interest in future work to evaluate how individual differences in maternal reward responding may relate to individual differences in some of the highlighted genes.

The magnitude of gene expression change observed in this study is consistent with those in other postpartum brain studies (Gammie et al., 2005; Xiao et al., 2005; Zhao et al., 2012b; Eisinger et al., 2013b, 2014; Driessen et al., 2014a), suggesting the findings are real and biologically significant. Further, in the previous array studies and here, the magnitude of change assessed using qPCR matches that of the array, suggesting a significant and real biological level of change. In recent work, we found protein level changes also to be significant and to match those of mRNA analysis for glutamic acid decarboxylase in lateral septum (LS; Zhao et al., 2012a) and for *Nr1d1* and *Fabp7* in multiple brain regions (unpublished observations). Although the changes may not appear as dramatic as those found using cell cultures, they are similar to a wide range of other studies on manipulations (e.g., knockout, drug treatment, exercise, or aging) in the CNS (Piechota et al., 2010; Kohman et al., 2011; Kõks et al., 2011). Thus, the 21% decrease in Nr1d1, a transcription factor, would be expected to have an important effect. Given the fine-tuned nature of the CNS, even a 10% change of a gene could be expected to have a biologically significant effect, but the effect of any change would need to be tested directly.

### Postpartum NAC genes involved in transcriptional regulation

One notable feature of postpartum NAC genes was the large number involved in transcriptional regulation. This was seen with both MSET analysis using a transcriptional database and with NIH DAVID pathway analysis. One-hundered and fifty-three genes with an array *p* < 0.01 are involved in transcriptional regulation (Supplementary Table 5). WGCNA analysis provides indirect information on which of these transcriptional regulators may be contributing most to altered expression of other genes. Interestingly, *Nr1d1* (Figure 6) was identified as a possible key regulator of other maternal genes such as *Penk* and *Grm3*. Other circadian transcriptional regulators, including *Cry1*, *Per1*, and *Per2* were part of a different module, but also suggested to regulate some postpartum genes. As also seen in Figure 6, a large number of postpartum genes were suggested to be regulated by a small subset of transcriptional regulators, including *Fosb*, *Jun*, *Egr1*, *Ar*, *Dmtf1*, *Nr4a3*, *Nr4a1*, *Ncoa7*, *Egr2*, *Hdac5*, and *Irf2*. Weighted Gene Coexpression Network Analysis provides a starting point for investigating how natural changes in NAC occur. Whether or to what extent the transcriptional modules produced by WCGNA reflect how the postpartum NAC is produced can be evaluated in subsequent studies.

### Genes with altered expression in postpartum NAC enriched for genes linked to mental health disorders

Postpartum NAC gene expression showed particularly high enrichment for BPD (three of three databases), schizophrenia (five of five databases), and depression (two of three databases). Autism showed modest enrichment (three of seven databases). Together, this profile is similar to that recently found for postpartum mPFC, whereby schizophrenia and BPD showed the strongest enrichment (Eisinger et al., 2014). One explanation for such enrichment is that the same genes that are actively regulated to produce a maternal phenotype are also ones that can be dysregulated in a mental health disorder. As an example, it could be possible that hundreds of genes have altered expression to elevate sociability and bonding in a mother, but if those same genes are dysregulated, then decreases in sociability or bonding could occur. For most mental health disorders, deficits in sociability are an endophenotype. Another view is that hundreds of genes are needed for the complex maternal phenotype, but if any of those changes go amiss, then a disorder could occur. This outlook is consistent with the finding of increased risk in mothers for depression, BPD, and postpartum psychosis (with connections to schizophrenia) (Brockington, 2004; Sit et al., 2006; Spinelli, 2009; Maina et al., 2014). Among genes of interest with high links to mental disorders are *Grm3*, *Fabp7*, and *Nr1d1*. Recent work suggests *Nr1d1* regulation of *Fabp7* expression (Schnell et al., 2014b) and as indicated above, a role for *Nr1d1* in addictive and reward related processes are now being established. Whether *Nr1d1* could play an integral role in the high rates of addiction in those with mental health disorders is an interesting, but untested hypothesis. How to interpret or understand the links of the maternal brain genes with those for mental health disorders will take time to determine, but knowledge of shared genes can help inform both our view of the maternal brain, but also of how mental health disorders occur.

### Enrichment of developmental processes within postpartum NAC

A number of postpartum NAC genes were found to be linked to CNS plasticity and development via NIH DAVID. These processes included axon growth, axonogenesis, neurogenesis, and neuron differentiation. The finding of enrichment for CNS plasticity and development is similar to our recent postpartum microarray findings for LS, medial preoptic area (MPOA) and mPFC (Eisinger et al., 2013b, 2014; Driessen et al., 2014a). These cumulative findings suggest the maternal brain represents a developmental endpoint and are consistent with previous work demonstrating CNS plasticity in lactating females (Gregg et al., 2007; Kim et al., 2010; Leuner and Gould, 2010; Lévy et al., 2011). Differentiation and plasticity can occur in the absence of neurogenesis or gliogenesis and it is possible that many of the postpartum CNS developmental events are occurring within intact cells as opposed to within newly generated cells. For example, decreases of *Fabp7* can lead to altered identity of cells expressing *Fabp7*, but also of neighboring cells (Owada, 2008; Boneva et al., 2011; Kipp et al., 2011; Mitchell and Hatch, 2011). The extent of CNS plasticity is likely quite large, but focused studies would be needed to help untangle how specific gene expression changes translate into function CNS changes.

### One model for the emergence of reward and addiction to offspring with a focus on opioids

As detailed in the Introduction section, a large body of work has highlighted the maternal state as involving and increases reward responding to offspring. Although we highlight numerous genes in NAC that may contribute to the maternal phenotype, some of these are particularly noteworthy. In postpartum NAC, both endogenous opioids, *Pdyn* and* Penk*, show highly significant decreases in expression. Further, these postpartum decreases of *Penk* and *Pdyn* are also found in mPFC and in LS (Eisinger et al., 2013b, 2014). Normally, the decreases of endogenous opioid signaling in NAC and associated regions would be associated with a decreased ability of reward responding (Kelley and Berridge, 2002; Koob and Volkow, 2010). Interestingly, MPOA has significant increase in *Penk* (Driessen et al., 2014a) and MPOA is considered a critical maternal brain region. One model, then, is that in the transition to motherhood there is a general decrease in reward (via decreased endogenous opioids) for other rewarding natural stimuli. However, because MPOA enkephalin is elevated, there is now a heightened rewarding effect of any maternally related signal. A role for endogenous opioids in maternal care has previously been suggested (Panksepp et al., 1994) and here a new insight may be that rewards from other signals are reduced in mothers, while rewards from offspring are enhanced. In earlier work we found elevated enkephalin in a large tissue region that included both hypothalamus and MPOA (Gammie et al., 2005). Likewise, it is possible that increases in *Penk* from feeding associated regions, such as arcuate nucleus, has the similar effect so that in the maternal state, offspring and eating are the primary forms of reward type signaling. Although this is speculative, it does provide a testable model for how a natural switch in reward and addiction can occur with a focus on opioids.

### Circadian rhythm and acetylation genes

Circadian rhythms are intertwined with multiple processes, including addictions and mental health disorders, for recent reviews, see Damaggio and Gorman (2014), Landgraf et al. (2014) and Schnell et al. (2014a). We found enrichment for circadian rhythm genes and those included: *Csnk1e*, *Cry1*, *Npas2*, *Nr1d1*, *Per1*, and *Per2*. *Nr1d1* is of particular interest because it is a transcription factor that interacts with clock proteins (Ueda et al., 2005). Also, *Nr1d1* has now been found to be reliably and significantly reduced in four maternal brain regions, NAC, mPFC, MPOA, and LS (Eisinger et al., 2013b, 2014; Driessen et al., 2014a), suggesting its downregulation is critical for production of the maternal brain. We also found enrichment for genes related to acetylation events and some of these may have relevance to a recent finding for involvement of deacetylation in supporting gene expression and maternal care in mice (Stolzenberg et al., 2014) and acetylation processes in gene expression and pair bonding in prairie voles (Wang et al., 2013).

### Concluding remarks

In this study, we evaluate for the first time large scale gene expression changes that occur in NAC in mothers when pups have become highly rewarding. Using bioinformatics tools we find enrichment for genes involved in addiction and reward using multiple independently curated databases. A novelty of this study is the identification of over a hundred addiction/reward-related genes that are likely recruited and promote the emergence of a new natural reward, the offspring. Interestingly, in rodents, the rewarding properties of pups begins waning about half way through lactation (Seip and Morrell, 2007), so the maternal rodent brain provides an example of how an animal can both increase and lessen a natural reward. Further, mothering decreases the rewarding properties of drugs (Mattson et al., 2001, 2003; Ferris et al., 2005), so it is possible that insight from the maternal NAC could provide new insights into how to mitigate addictions.

## Conflict of interest statement

The authors declare that the research was conducted in the absence of any commercial or financial relationships that could be construed as a potential conflict of interest.
